# Towards Detecting Red Palm Weevil Using Machine Learning and Fiber Optic Distributed Acoustic Sensing

**DOI:** 10.3390/s21051592

**Published:** 2021-02-25

**Authors:** Biwei Wang, Yuan Mao, Islam Ashry, Yousef Al-Fehaid, Abdulmoneim Al-Shawaf, Tien Khee Ng, Changyuan Yu, Boon S. Ooi

**Affiliations:** 1 Computer, Electrical and Mathematical Sciences and Engineering (CEMSE) Division, King Abdullah University of Science and Technology (KAUST), Thuwal 23955-6900, Saudi Arabia; biwei.wang@kaust.edu.sa (B.W.); yuan.mao@kaust.edu.sa (Y.M.); islam.ashry@kaust.edu.sa (I.A.); tienkhee.ng@kaust.edu.sa (T.K.N.); 2 Department of Electronic and Information Engineering, The Hong Kong Polytechnic University, Hong Kong, China; changyuan.yu@polyu.edu.hk; 3 Center of Date Palms and Dates, Ministry of Environment, Water and Agriculture, Al-Hassa 31982, Saudi Arabia; alfehaid2000@gmail.com (Y.A.-F.); yassir1418@yahoo.com (A.A.-S.)

**Keywords:** red palm weevil, fiber optic acoustic sensing, machine learning

## Abstract

Red palm weevil (RPW) is a detrimental pest, which has wiped out many palm tree farms worldwide. Early detection of RPW is challenging, especially in large-scale farms. Here, we introduce the combination of machine learning and fiber optic distributed acoustic sensing (DAS) techniques as a solution for the early detection of RPW in vast farms. Within the laboratory environment, we reconstructed the conditions of a farm that includes an infested tree with ∼12 day old weevil larvae and another healthy tree. Meanwhile, some noise sources are introduced, including wind and bird sounds around the trees. After training with the experimental time- and frequency-domain data provided by the fiber optic DAS system, a fully-connected artificial neural network (ANN) and a convolutional neural network (CNN) can efficiently recognize the healthy and infested trees with high classification accuracy values (99.9% by ANN with temporal data and 99.7% by CNN with spectral data, in reasonable noise conditions). This work paves the way for deploying the high efficiency and cost-effective fiber optic DAS to monitor RPW in open-air and large-scale farms containing thousands of trees.

## 1. Introduction

**Figure 1:**
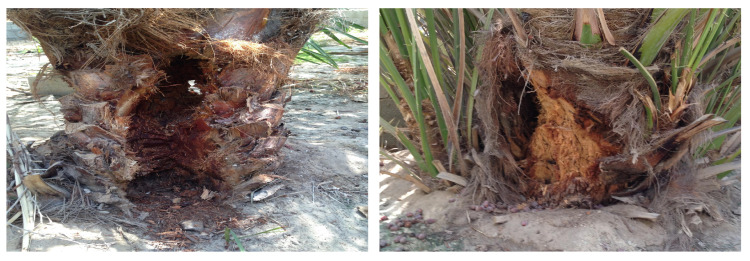
Two representative examples of treated trees by scraping to remove red palm weevil (RPW).

The date palm is a high-value fruit crop that provides healthy nutrition security to millions of people around the world [1]. It is further considered an important source of export revenue for rural smallholders worldwide. Unfortunately, the date production and trade are in danger because of the red palm weevil (RPW), also named as Rhynchophorus ferrugineus [2,3]. RPW is a Coleopteran snout pest, which is considered the single most destructive pest of palm trees. Young and soft trees aged less than 20 years, which represent ∼50% of the total cultivated date palm trees, are vulnerable since RPW typically targets them [4]. Beside date palms, RPW also attacks coconut, oil, and ornamental palms [4,5]. In the past few decades, RPW has been found in more than 60 countries including the Mediterranean region, parts of Central America, Middle East, North Africa, among others [4,6]. This plague has globally destroyed many palm farms causing severe economic losses in a form of lost production or pest-control costs. As representative examples, Figure 1 shows the RPW’s impact on two date palm trees, after treating the trees with scraping to entirely remove the RPW.

In the early stage of infestation, palm trees can be healed with chemical treatments [7]. However, a palm tree only shows visual distress signs in a well-advanced stage of infestation, where it is difficult to save the tree. Many techniques have been reported in the literature for the early detection of RPW [8,9,10]. Some detection methods such as x-ray based tomography [9] and trained dogs [10] are accurate; however, they lack the feasibility in large-scale farms because of their slow scanning processes. The most promising early detection methods are based on sensing the larvae sound, while they are chewing the core of a palm trunk. The larvae start to produce eating sound in an early infestation stage, where the larvae are less than two weeks old [11]. Existing acoustic detection technologies rely on inserting acoustic probes within the individual tree trunks and building a wireless network to communicate with the sensors [8]. Unfortunately, it is cost-ineffective to assign a sensor per tree, especially for vast farms containing thousands of trees. Additionally, this method is invasive and may harm the trees or create nests for insects.

We recently reported a solution of using a fiber optic distributed acoustic sensor (DAS) for the early detection of RPW, such that a single optical fiber is noninvasively wound around the palm trees to possibly scan a large-scale farm within a short time [11,12]. As reported in [11], distinguishing the healthy and infested trees was achieved through a straightforward signal processing algorithm since the experiment was carried out in a controlled environment. Identifying infested trees in open-air farms, where the optical fiber might be subjected to harsh environmental noises, would require a more advanced signal processing technique to classify the larvae sound and other noise sources.

To pave the way for utilizing the fiber optic DAS to monitor real farms, here, we introduce neural network-based machine learning algorithms to classify healthy and infested trees, based on the data collected by a fiber optic DAS. Within a laboratory environment, we mimic the environmental conditions of a farm that includes the healthy/infested palm tree and other noise sources. In particular, a sound of ∼12 day old weevil larvae is played inside a tree trunk and meanwhile, the tree is subjected to external wind and bird sounds as noise sources. A fully-connected artificial neural network (ANN) [13] and a convolutional neural network (CNN) [14] are used to recognize the infested and healthy trees in the noisy environment. We further investigate the impact of different optical fiber’s jackets on mitigating the external noise around the trees. This work would be highly beneficial towards the future deployment of the fiber optic DAS for the early detection of RPW in vast real farms.

## 2. Experimental Design

**Figure 2:**
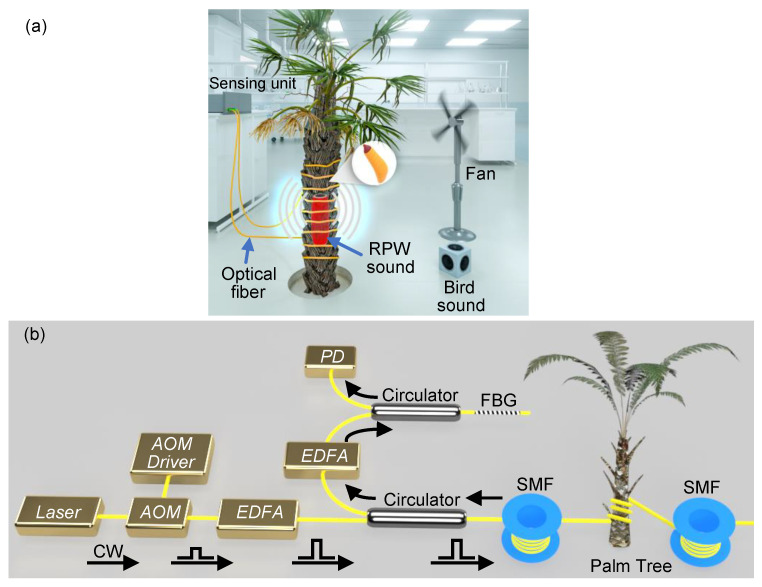
(**a**) Overall design of using the fiber optic distributed acoustic sensor (DAS) to detect the RPW sound. (**b**) Experimental setup of the DAS interrogation unit.

Figure 2a shows the overall design of our experiment. The optical and electronic components of the DAS system are assembled within a sensing unit, whose contents will be described shortly later. The output light from the sensing unit is launched into a single-mode fiber (SMF) and we wind a section of the fiber around a tree trunk. Within the trunk, we implant a loudspeaker (SRS-XB21, Sony, 20 Hz–20,000 Hz frequency transmission range) that continuously plays an eating sound of ∼12 day old weevil larvae. At a ∼1 m distance from the tree, there is a fan that blows air, with a speed of ∼3 m/s, towards the optical fiber and tree. Additionally, also at a ∼1 m distance from the tree, we locate another loudspeaker (AudioCube, Allococac, 40 Hz–20,000 Hz frequency transmission range) that continuously produces bird sounds. At the outer surface of the tree, we place a sound level meter that can record the various sound intensity levels used in the experiment. The measured sound intensity level of the background noise within the laboratory is ∼51 dB, caused by the instruments working in the laboratory, which raises to ∼71 dB when only playing the bird sounds. The intensity level of the bird sounds is roughly equal to that we hear in farms. According to the literature [8] and also our experience, humans can hear the larvae sound under acceptable environmental noise. As measured by the meter, when only the loudspeaker within the tree is turned on, we set the intensity levels of the larvae sound to be within the range [∼51 dB–∼75 dB]. The low-level cannot be distinguished from the background noise by the meter, while the other high-threshold is obvious. The selected larvae sound’s intensity levels within this range should represent all possible degrees of infestation (weak, medium, and strong infestation).

The sensing unit comprises the interrogation system of the fiber optic DAS (Figure 2b), which is designed using the phase-sensitive optical time-domain reflectometry (ϕ-OTDR) [15]. A narrow linewidth laser generates a continuous wave (CW) light of a 100 Hz linewidth and a 40 mW optical power. The laser light is then converted into optical pulses using an acousto-optic modulator (AOM) that produces pulses of a 50 ns width and a 5 kHz repetition rate. The selected pulse width offers a DAS system of a 5 m spatial resolution. The power of the optical pulses is amplified using an erbium-doped fiber amplifier (EDFA), while its output light is launched through a circulator into a standard SMF of a ∼2 km length. At a ∼1 km distance from the input port of the SMF, we wind a 5 m section of the fiber around the tree trunk. The backscattered signal from the SMF is amplified with another EDFA, which amplified spontaneous emission (ASE) noise is discarded using a fiber Bragg grating (FBG). The filtered Rayleigh signal is detected by a photodetector (PD) and sampled by a digitizer of a 200-MHz sampling rate. Finally, the Rayleigh signals are recorded as 1-s periods (5000 traces per period). This experiment includes utilization of two separate standard SMFs, protected with different jackets of a 900 μm diameter (Thorlabs, SMF-28-J9, denoted as “JKT1”) and a 5 mm diameter (YOFC, YOFC-SCTX3Y-2B1-5.0-BL, denoted as “JKT2”), respectively.

**Figure 3:**
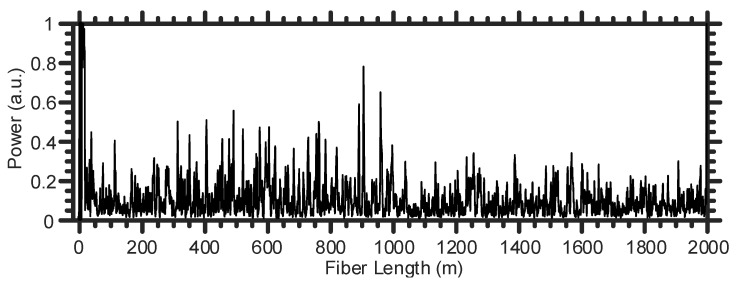
A representative example of a Rayleigh trace recorded by the fiber optic DAS.

Figure 3 shows an example of a Rayleigh trace recorded by the fiber optic DAS system. The high-power signal located at the start of the SMF is typical and it corresponds to the Fresnel reflection from the front facet of the SMF. In the ideal scenario, when there is no refractive index perturbation along the SMF, the shape of the Rayleigh trace remains stationary in the time-domain for all the spatial points along the entire fiber [15,16]. Consequently, the differences between the temporal subsequent Rayleigh traces and an initial reference one would be ideally zeros. In contrast, the presence of a larvae sound within the tree trunk can modulate the fiber’s refractive index at the tree position, which results in changing the corresponding temporal Rayleigh signal only at the tree location. By applying the normalized differential method [17] and fast Fourier transform (FFT) to the temporal Rayleigh traces, the location of an infested tree and the larvae sound’s frequencies can be identified, respectively.

## 3. Investigating the Impact of the Noise Sources on the DAS System

In this section, we explore the possible ways of mitigating the environmental noises, such as wind and bird sounds, which may degrade the performance of the fiber optic DAS system when detecting the RPW. The suggested techniques of reducing the noise include applying a spectral band-pass filter to alleviate the noise level within the recorded signals and further trying various optical fiber’s jackets which might be shaken because of the wind. This investigation is necessary not only to improve the performance of the machine learning algorithms during classifying the healthy and infested trees but also to make the DAS system more feasible for real RPW detection.

**Figure 4:**
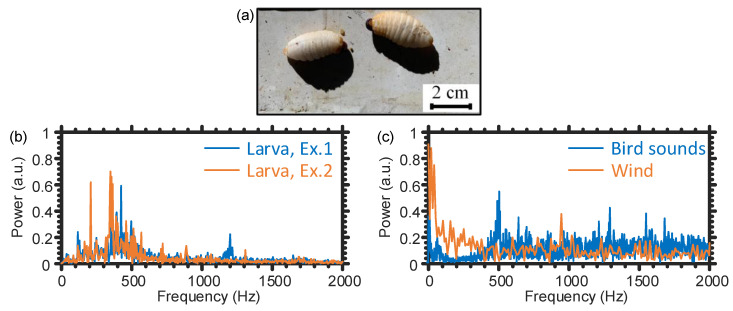
(**a**) ∼12 days old weevil larvae. (**b**) Two representative examples of the larvae sound’s spectra, marked as Ex. 1 and Ex. 2, produced using the data of the voice recorder. (**c**) Examples of the power spectra produced by bird sounds and wind.

Firstly, the spectral components of the actual larvae sound are explored. In particular, a commercial voice recorder (ICD-UX570, Sony, 50 Hz–20,000 Hz frequency response) is implanted inside a truly infested tree trunk and next to ∼12 day old larvae, shown in Figure 4a, such that the voice recorder stores the larva’s sound using the uncompressed linear pulse-code modulation (LPCM) format to always have a pristine quality audio file [18]. We select this specific RPW life stage to examine if our sensor can detect the larvae sound at an early stage, so that the palm tree can still be saved and cured. During the recording time, the larvae are eating and moving naturally within the trunk without any restriction. Consequently, the quality of the simulated sound in the laboratory should be comparable to the real one. The age of the larvae can be well controlled via an artificial infestation process [11], where it is carried out in a secured research facility to avoid spreading the RPW to other healthy trees. Interestingly, it is observed that the larvae almost continuously produce the sound while they are chewing the tree trunk. Figure 4b shows two different representative examples of the larvae sound’s power spectra, where each corresponds to a 0.5 s recording interval. The results of Figure 4b indicate that the majority of the larvae sound’s optical power is around 400 Hz.

In contrast, the used bird sounds have a broad spectrum (Figure 4c, blue line), which interferes with that of the larvae. Regarding the wind as a noise source, the orange line in Figure 4c represents an example of the vibration’s power spectrum caused by wind when shaking the “JKT2” fiber. The vibration caused by wind is dominated by the tree swinging, which has low-frequency components. However, wind may also directly shake the optical fiber to produce other vibrations of high frequencies, which may overlap with that of the larvae sound. This is because the vibration strength caused by the wind is larger than that of the larvae sound; therefore, the wind’s high-frequency vibration has to be taken into account as a noise source. Given the results of Figure 4b,c, for the entire following temporal vibration data that we collect using the fiber optic DAS, we will apply a [200 Hz–800 Hz] band-pass filter to enhance the signal-to-noise ratio (SNR) of our system. This is because the spectral filter can discard the low vibration frequency components, less than 200 Hz, to cancel the inevitable mechanical vibration in the laboratory and the tree swinging caused by wind. Meanwhile, it filters out the high-frequency (larger than 800 Hz) components, produced by the electronic/optical components in the system, without impacting the larvae sound’s dominant frequencies (around 400 Hz).

**Figure 5:**
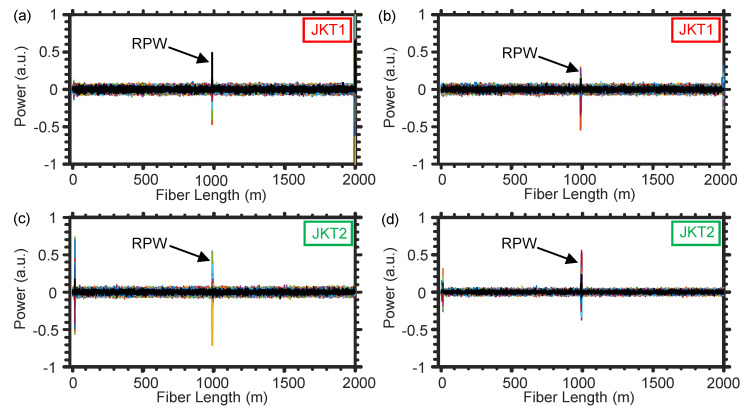
Representative examples of the infested tree’s position information when using the SMF of JKT1 (**a**,**b**) and JKT2 (**c**,**d**).

Focusing on the experimental design of Figure 2a, we initially switch off the fan and the outside noise loudspeaker, while we only play the larvae sound using the loudspeaker implanted inside the tree trunk. Figure 5a–d show two representative examples of the normalized differential time-domain signals recorded using the DAS system [17], followed by applying the [200 Hz–800 Hz] band-pass filter, when using the SMF of JKT1 [JKT2]. Clearly, the two fibers accurately locate the position of the infested tree at the ∼1 km distance from the input ports of the fibers. The other noisy signals, which sometimes appear at the start of the SMFs, are a result of the fiber front facet’s reflection.

**Figure 6:**
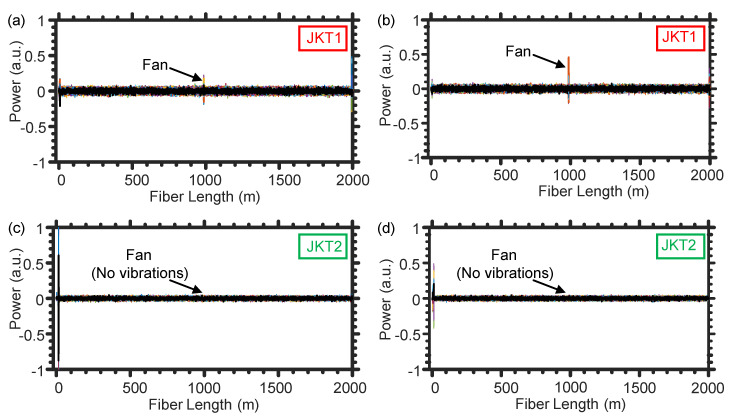
Representative examples of the temporal vibrations caused by the wind when using the SMF of JKT1 (**a**,**b**) and JKT2 (**c**,**d**).

Next, the two loudspeakers are switched off and we only turn on the fan to inspect the impact of the wind on the two SMFs. Wind would be considered the primary noise source in open-air farms, especially because our detection technique is noninvasive and the fiber would typically be subjected to vibrations caused by wind. Even with applying the [200 Hz–800 Hz] band-pass filter, the SMF of JKT1 is impacted by the wind to produce temporal vibrations as those shown in Figure 6a,b. The low frequency vibrations, produced by tree swinging as a result of the wind, can be easily discarded with filtering out the frequencies below 200 Hz [19]. However, blowing the wind to directly hit the optical fiber results in shaking the fiber with frequencies that rely on the thickness and material of the fiber’s jacket. As shown in Figure 6a,b, the SMF of JKT1 that has a relatively small diameter (900 μm) produces vibration signals, caused by the wind, which may resemble those of the larvae sound. This behavior may confuse the machine learning algorithms during distinguishing the healthy and infested trees.

Similarly, while switching off the two loudspeakers and turning only the fan on, we use the DAS system when winding the SMF of JKT2 around the tree trunk. Since the JKT2 is relatively thick (5 mm diameter), the fiber rarely generates shaking frequencies within the [200 Hz–800 Hz] range because of the wind [Figure 6c,d]. Such comparison between the two fiber jackets in terms of mitigating the noise produced by wind is crucial for determining the proper optical fiber cable that can be used in the future in real farms. Besides, compared with JKT1, JKT2 has an additional advantage that it is durable enough to sustain the harsh environmental conditions of farms and the SMF inside JKT2 cannot be easily broken by, for example, stepping on the fiber by farmers.

**Figure 7:**
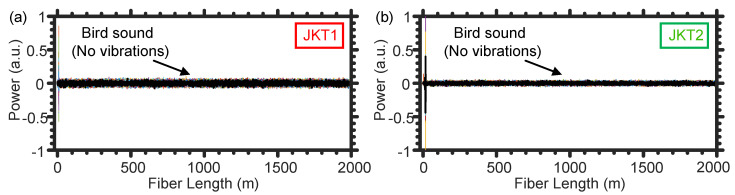
Representative examples of the temporal vibrations caused by the bird sounds when using the SMF of JKT1 (**a**) and JKT2 (**b**).

We further investigate the impact of the noise produced by bird that may surround the optical fiber in farms. In particular, we switch off the larvae sound’s loudspeaker and the fan, while playing only the outside loudspeaker. Fortunately, the two SMFs of JKT1 and JKT2 cannot “hear” the bird sounds, as shown respectively in Figure 7a,b. This is because the air between the loudspeaker and the optical fiber jackets significantly attenuates the vibration energy of the bird sounds. Typically, a fiber optic DAS system can be used to sense sound propagating through the air by utilizing a thin metallic sheet, attached to the fiber, to amplify that attenuated vibration energy by air [20]. In our experiment, however, we do not use a metallic sheet to avoid recording the acoustic noise signals generated around the tree.

It is also worth discussing the impact of using the JKT1 and JKT2 on the overall noise floor. The noise floor depends on many factors such as temporal pulse intensity fluctuation, laser phase noise and frequency drift, low extinction ratio of the launched pulses, photodetector thermal, and shot noise [15,21], which are all common when using the JKT1 or JKT2. However, another major factor that contributes to the noise floor is the overall isolation of the optical fiber from externally induced vibrations. Consequently, the thicker jacket (JKT2) typically provides a lower noise floor.

## 4. Classifying Infested and Healthy Trees Using Machine Learning Methods

Machine learning methods trained through supervised learning can be effective approaches for identifying the infested and healthy trees. Machine learning can reveal patterns associated with the larvae sound and simultaneously deal with the large amount of data produced by the DAS system. In this work, we compare the efficiencies of classifying the healthy and infested trees when using the time- and frequency-domain data as separate inputs to neural networks, which are designed using the fully-connected ANN and CNN architectures. Given the aforementioned advantages of the SMF of JKT2, we decide to use it in the subsequent analyses of classifying the healthy and infested trees using machine learning methods.

We initially focus on the way of organizing and labeling the time- and frequency-domain data for the ANN. As aforementioned, we wind a 5 m section of the fiber around the tree, while the digitizer is sampling the data at a 200 MHz frequency. Consequently, given the time-of-flight within the OTDR sensing system, the optical fiber section around the tree is represented by 10 spatial points, i.e., the digitizer’s sampling resolution is ∼0.5 m. For each point, the digitizer reading takes a 1 s period, i.e., 5000 readings in the time-domain per one reading period because the pulse repetition rate is 5 kHz. Since the digital band-pass filter typically distorts a short-interval at the beginning of the time-domain signal, we discard the first 250 time-domain readings for each spatial point. Thus, the collected temporal data in each trial are organized as a vector of 47,500 length (concatenating 4750 time-domain readings ×10 spatial points). In contrast, by applying the FFT to the time-domain data of each spatial point, we get 2375 frequency components. Subsequently, we organize the spectral data of each trial as a vector of 23,750 length (concatenating 2375 frequency components ×10 spatial points).

We label the data as “infested” or “healthy” tree, based on the SNR value of the acoustic signal at the tree position. We define the SNR as the ratio between the root-mean-square (RMS) value of the time-domain signal at the tree position and that at another calm reference fiber section of a 5 m length. We evaluate the ability of the machine learning algorithms to classify the infested and healthy trees in two cases, without and with the presence of wind. Considering the first case when ignoring the wind, we play the loudspeaker within the tree trunk and stop the fan to mark the signals of the infested tree. If the SNR > 2 dB, the minimum acceptable SNR of a DAS system [17], we record and label the signal as “infested”. We collect 2000 examples of the infested signals, when the sound of the larvae loudspeaker is set at various intensity levels within the aforementioned [∼51 dB–∼75 dB] range. In contrast, we record other 2000 samples for the “healthy” signals, when the larvae loudspeaker and fan are off. The “healthy” signal examples are recorded regardless of the SNR value to be higher or lower than the 2 dB threshold.

Focusing on labeling the data when considering the presence of the wind, we turn on simultaneously the larvae loudspeaker and the fan to record the examples of the “infested” signals. Similarly, we record 2000 various examples when the SNR values exceed the 2 dB threshold. Next, we switch off the larvae speaker while keeping the fan on in order to record the other 2000 examples, regardless of the SNR values, for the healthy tree.

**Figure 8:**
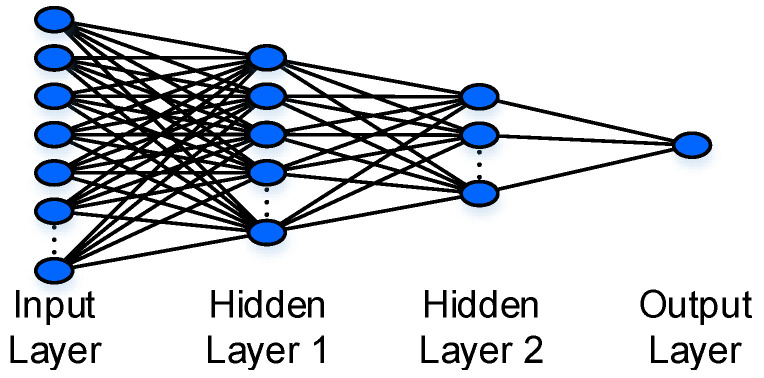
The ANN structure for detecting the RPW infestation using the temporal/spectral data.

The ANN models used to handle the time- and frequency-domain data have a similar architecture, which is shown in Figure 8. This structure consists of one input layer, two hidden layers, and one output layer. The number of nodes in the input layer matches the number of elements in the data vectors, i.e., 47,500 and 23,750 for the time- and frequency-domain data, respectively. Besides, the first and second hidden layers respectively comprise 500 and 50 nodes, determined by repeated and sufficient trials to maximize the classification accuracy. At the end of the fully-connected ANN, there is an output layer of one node for the binary classification (infested or healthy). Regarding the activation functions, we use the rectified linear unit (ReLU) for the hidden layers and the sigmoid function for the output layer.

**Figure 9:**
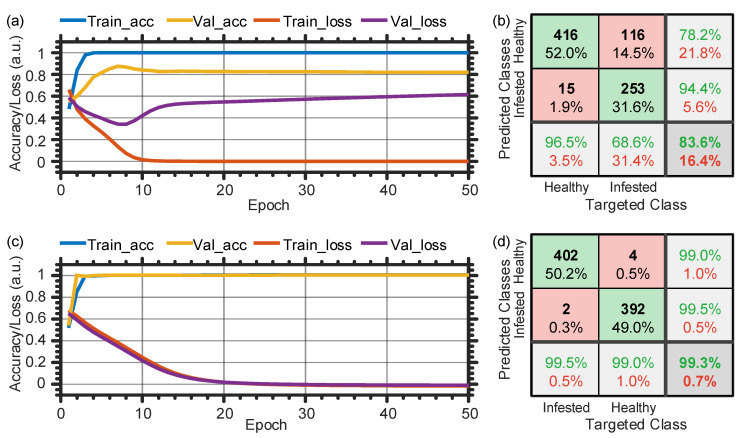
Training and validation history (**a**/**c**) and confusion matrix (**b**/**d**) when ignoring the wind and using the temporal/spectral dataset with the ANN. Train_acc: training accuracy; Val_acc: validation accuracy; Train_loss: training loss; Val_loss: validation loss.

When the wind is ignored (the fan is turned off), we split the collected temporal/spectral data as 60% (2400 examples) training, 20% (800 examples) validation, and 20% (800 examples) testing datasets. In this scenario, Figure 9a,c shows the evolution of the training/validation accuracy and loss with the epoch, when using the temporal [spectral] data. At the end of the training cycles, validation accuracy values of 82.0% and 99.8% are produced for the time- and frequency-domain data, accordingly. When using the temporal data [Figure 9a], the final validation accuracy is obviously lower than that of the training process, which indicates the model cannot be generalized. In contrast, as shown in Figure 9c of the spectral data, the validation accuracy perfectly matches with the training one, which confirms that the ANN model learns the features well, instead of just remembering the input data.

Following the training and validation processes, we use the testing datasets to estimate the performance of the two models. Figure 9b,d show the confusion matrices when using the time- and frequency-domain data, respectively. In general, a confusion matrix comprises four main indices denoted as true negatives (TN), false negatives (FN), false positives (FP), and true positives (TP), which compare the actual target values with those predicted by the machine learning model [22]. Besides, some other important performance metrics (accuracy, precision, recall, and false alarms) are also included in the confusion matrix and defined as [22]:

$$\begin{array}{ccc} Accuracy & = & (TP+TN)/(TP+FP+TN+FN),\\ Precision & = & TP/(TP+FP),\\ Recall & = & TP/(TP+FN),\\ FalseAlarm & = & FP/(TP+FP). \end{array}$$

As shown in the confusion matrices of Figure 9b,d, the temporal data provides a total classification accuracy of 83.6%, while that of the spectral data is 99.3%. The entire ANN’s performance parameters are summarized in Table 1, first and second rows, when neglecting the wind and using the temporal and spectral data. As a result, to get a high distinguishing accuracy between the infested and healthy trees, it is recommended to use the ANN with the spectral data of the larvae sound. This is attributed to that the chewing sound of the larvae can be shifted within the 1 s recording frame, which makes it difficult for the ANN model to learn using a limited dataset. However, the shifted temporal acoustic signals produce similar spectra, which facilitate the classification process using the frequency-domain data. Given this conclusion, we decide to rely on the spectral components with the ANN to analyze the subsequent more complex scenario, when the wind impact is considered.

We split again the spectral data, collected when the fan is turned on, as 60% (2400 examples) training, 20% (800 examples) validation, and 20% (800 examples) testing datasets. After the training and validation processes, the spectral testing dataset is used to examine the performance of the trained model. For this scenario, the third row of Table 1 shows a summary of the ANN’s performance results. The ANN model provides a total classification accuracy of 99.6%, which is slightly higher than that produced in the case of neglecting the wind. The precision, recall, and false alarm rates also show minor improvements. These results indicate that the ANN model can perfectly learn the larvae sound’s spectral pattern in the two scenarios, without and with wind, while the tiny perturbations caused by the wind slightly increases the robustness and generalization ability of the model.

A more realistic case to consider is combining the two spectral datasets, with and without the wind as a noise source. This is reasonable since the air blows intermittently in real farms. Thus, we merge the two datasets to have in total 8000 examples for the infested and healthy trees. Again, we split the entire data as 60% (4800 examples) training, 20% (1600 examples) validation, and 20% (1600 examples) testing datasets. When using the combined data, the classification accuracy, precision, recall, and false alarm rates are improved [fourth row of Table 1], as compared with those of the two former separate cases. These results indicate that the performance of the ANN model is enhanced given the large quantity and more variety of the training data. Thus, one can conclude that the ANN model performs excellently when using the combined spectral data of the more realistic scenario in farms; however, the ANN model has a relatively poor performance with the temporal input data.

**Figure 10:**
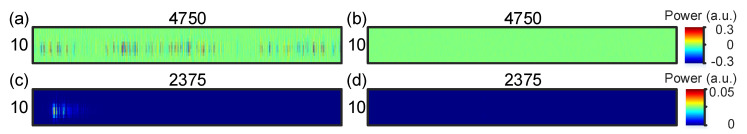
CNN model’s input images of the (**a**) temporal and “infested”, (**b**) temporal and “healthy”, (**c**) spectral and “infested”, and (**d**) spectral and “healthy” data.

CNNs are popular deep neural network structures, designed to be spatially invariant [14]. In other words, they are not sensitive to the position of the features, which would be effective in handling the temporal larvae sound that is shifting in the time-domain. In addition, compared with the fully-connected ANNs, CNNs have relatively less parameters to train, which makes CNNs easier and more efficient to train with the same quantity of datasets [14,23]. Since CNNs have proven high efficiency in classifying images, we arrange the temporal and spectral data in two-dimensional matrix forms. In particular, the time- and frequency-domain examples are arranged as 10 (spatial points) × 4750 (temporal readings) and 10 (spatial points) × 2375 (spectral components), respectively. As representative examples, Figure 10a–d show the CNN model’s input images of the (a) temporal and “infested”, (b) temporal and “healthy”, (c) spectral and “infested”, and (d) spectral and “healthy” data, respectively.

**Figure 11:**
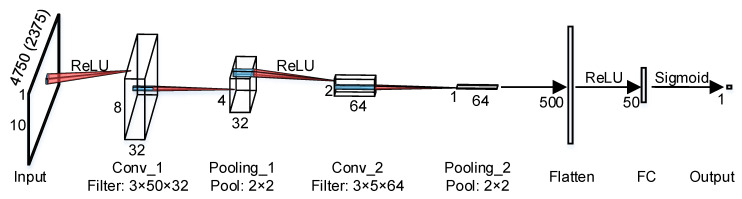
The CNN structure for detecting the RPW infestation using the temporal/spectral data. Conv: convolutional; FC: fully-connected.

Figure 11 shows the architecture of the CNN model used to separately handle the temporal and spectral input data. The architecture respectively comprises an input layer, two pairs of convolutional and max pooling layers, a flatten layer, a fully-connected layer, and an output layer. The first (second) convolutional layer has the ReLU activation function and includes 32 (64) filters of a 3 × 50 (3 × 5) size and 1×25 (1 × 5) stride. The two max pooling layers have the same 2 × 2 pool size and 2 × 2 stride. After the flatten layer, the fully-connected layer has the ReLU activation function and consists of 50 nodes. Similar to the ANN, the output layer of the CNN also has one node with sigmoid activation function for the purpose of binary classification (healthy or infested).

**Figure 12:**
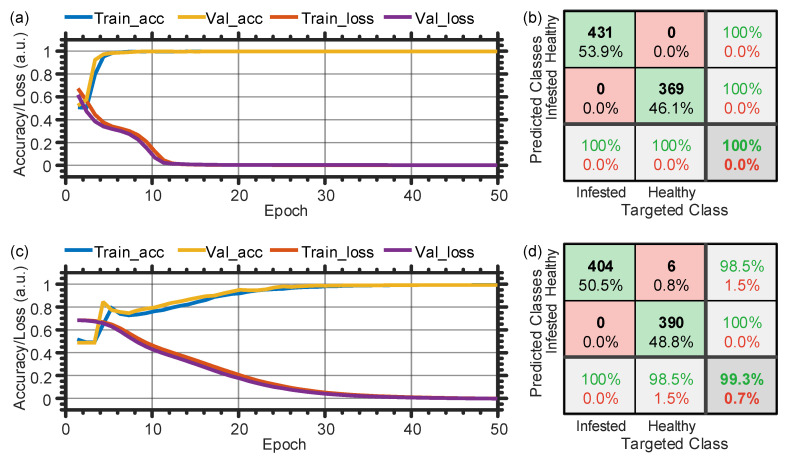
Training and validation history (**a**/**c**) and confusion matrix (**b**/**d**) when ignoring the wind and using the temporal/spectral dataset with the CNN. Train_acc: training accuracy; Val_acc: validation accuracy; Train_loss: training loss; Val_loss: validation loss.

In terms of the data labeling and splitting for the CNN model, we adopt the same techniques and data quantity as those used with the fully-connected ANN. Considering the ideal scenario when the wind is ignored (the fan is turned off), Figure 12a,c show the evolution of the training/validation accuracy and loss with the epoch for the temporal and spectral data, respectively. After finishing the training cycles, validation accuracy values of 100% and 99.5% are accordingly obtained when using the time- and frequency-domain data. Besides, the two confusion matrices when using the temporal and spectral testing datasets are shown in Figure 12b,d, respectively. The results of the confusion matrices show that the performance of the CNN with the temporal data is excellent with 100.0% accuracy, while that of the spectral data is slightly lower (99.3%). Clearly, as compared with the results of Figure 9, the CNN significantly improves the classification efficiency in the time-domain. This proves the aforementioned two main advantages of the CNN model over the fully-connected ANN model, i.e., the CNN’s spatial invariance and less parameters to train. These results are important since using CNN would offer a real-time detection of RPW, without the need to apply the intensive FFT to the time-domain data.

Table 2 summarizes the CNN’s performance when using the temporal and spectral data, in case of ignoring or considering the wind, or mixing the two scenarios. As can be observed, the CNN model has a superior performance in the various situations with a minimum classification accuracy of 98.3%. Taking into consideration that the time-domain data is easier to process, compared with the spectral data that require additional FFT step, we recommend using the CNN and time-domain data for the feasible detection of RPW. Given this conclusion and considering the more reasonable case of the combined data, the CNN with the temporal data provides 99.7% accuracy, 99.5% precision, 99.9% recall, and 0.5% false alarm [third row, Table 2]. The high precision and low false alarm values confirm the reliability of the CNN model in classifying the healthy and infested trees. On the other side, the high recall value represents the great ability and sensitivity of the CNN model to locate the “infested” signals from a mixed “healthy” and “infested” set.

## 5. Discussion

We have examined the possibility of using machine learning and fiber optic DAS to distinguish healthy and infested trees, in the laboratory environment. In farms, however, many financial and technical issues have to be considered during the practical implementation. As reported in the literature [24], the sensing range of the fiber optic DAS can be typically extended to ∼10 km with a spatial resolution down to 1 m. Assuming the separation between two consecutive trees is ∼10 m and we wind a ∼1 m fiber section around each tree, a DAS sensing unit can simultaneously monitor ∼1000 trees. The entire cost of our DAS system including the optical fiber cable is ∼US$37,000; thus, the monitoring cost per tree is ∼US$37. A recommended deployment plan comprises permanent installation of the optical fiber cables in farms, because fiber optics are relatively cheap and easy to be plugged in/out of the DAS unit, while sharing a portable DAS sensing unit between the farms. Such a plan can significantly reduce the monitoring cost per tree, as the 5 m fiber section, wound around a tree in our experiment, costs only ∼US$2. Alternatively, if the farms are close to each other, time-division-multiplexing (TDM) [25] could be adopted by connecting the farms’ optical fibers with a single sensing unit via an optical switch, to scan the individual farms in different time frames.

Besides, in a real palm tree farm, the optical fiber cable might be broken because of the farming activities around the trees. Fortunately, the SMF (YOFC, YOFC-SCTX3Y-2B1-5.0-BL) is well protected with metallic rods to make the fiber shockproof. Furthermore, the outer jacket layer of the fiber optic cable can sustain temperatures as high as 60 °C, which helps the fiber to “survive” in the farms’ harsh environments. For more protection, we recommend burying the optical fiber in the soil between the trees. In the worst-case scenario, if the fiber is getting broken for any reason, the OTDR system can accurately locate the fault point with the spatial resolution of the system [26]. A portable fusion splicer can be easily used to fix the optical fiber on-site.

In case of adopting the spiral winding of the optical fiber around trees in a farm, an annual maintenance to the sensing system would be needed because of the trees’ girth growing. Planning ahead for having redundant fiber lengths between trees will help readjust the fiber wraps around the trees later as they grow. Alternative to the spiral winding plan, longitudinal zigzag attachment of the fiber to the tree trunk is a backup strategy; however, a heavy-duty and stretchable plastic wrap would be used as an outer layer to provide sufficient contact between the optical fiber and tree trunk. The latter strategy is part of our plans for future work on this topic.

It is also worth discussing the conditions of contact between the fiber optic cable and the tree trunk. The spatial resolution of our fiber optic DAS system is 5 m, which indicates the system cannot distinguish the spatial separations between vibration events that occur within the 5 m distance. In case of winding a fiber length shorter than 5 m around the tree, vibration events occurring close to the tree may not be distinguished from those at the tree, resulting in generating false alarms. Thus, the minimum length of fiber needed in this experiment is 5 m. Besides, in our experiment, we wind a 5 m fiber optic section around the tree with moderate tightness. We observed that some points along the fiber section are not directly touching the tree trunk, because of the sharpness of the trunk at some locations; however, the DAS system still efficiently detects the larvae sound. In other words, having all the points along the 5 m fiber optic section in direct and firm contact with the trunk is not necessary to make the system works. In general, the more tightly we wind the fiber optic around the tree, the higher the SNR. However, it is experimentally difficult to quantify the relationship between the SNR and the tightness of winding the optical fiber. Additionally, winding a fiber section of length longer than 5 m would improve the performance of the DAS system. However, the 5 m spatial resolution we use already provides excellent classification accuracy.

This discussion shows that the main advantage of our sensor, compared with those reported in the literature, is that the fiber optic DAS provides distributed detection of RPW. Within a short time, an entire farm would be scanned, which saves time and effort as compared with the other detection methods [8,9,10] that inspect the trees individually. However, a drawback of our reported system is that the initial installation of the optical fiber requires time and effort, especially in vast farms. Fortunately, the fiber installation only needs to be carried out once per farm, and then the fiber can remain in the farm permanently.

## 6. Conclusions

Fully-connected ANN and CNN are used to classify an infested tree with RPW and a healthy tree, using the data provided by a fiber optic DAS. To mimic the farm environment within the laboratory, the weevil larvae sound is played inside a tree trunk, while wind and bird sounds are used as noise sources around the tree. Considering the common scenario when the wind blows discretely, the ANN performs perfectly with the frequency-domain data to offer a 99.9% classification accuracy. In contrast, for the same conditions of wind, the CNN produces 99.7% and 99.1% classification accuracy when using the temporal and spectral data, respectively. Although the CNN’s performance is excellent with both kinds of data, we recommend using the CNN with the temporal data to avoid the intensive FFT calculations, required to get the spectral components. The results of this work are significantly beneficial for the next step of deploying the fiber optic DAS for the early detection of RPW in real farms. 

## Figures and Tables

**Table 1:** Summary of the ANN’s performance for the various environmental conditions.

Data	Accuracy	Precision	Recall	False Alarm
Temporal data, without wind	83.6%	94.4%	68.6%	5.6%
Spectral data, without wind	99.3%	99.5%	99.0%	0.5%
Spectral data, with wind	99.6%	99.7%	99.5%	0.3%
Spectral data, combined	99.9%	99.9%	99.9%	0.1%

**Table 2:** Summary of the CNN’s performance for the various environmental conditions.

Data	Accuracy	Precision	Recall	False Alarm
Temporal data, without wind	100.0%	100.0%	100.0%	0.0%
Temporal data, with wind	99.9%	99.7%	100.0%	0.3%
Temporal data, combined	99.7%	99.5%	99.9%	0.5%
Spectral data, without wind	99.3%	100.0%	98.5%	0.0%
Spectral data, with wind	98.3%	99.5%	97.0%	0.5%
Spectral data, combined	99.1%	99.7%	98.3%	0.3%

## Data Availability

The data presented in this study are available on reasonable request from the corresponding author. The data are not publicly available due to privacy.
